# Diagnostic value of combined parameters derived from ambulatory electrocardiography for detecting coronary artery disease in non-active chest pain patients

**DOI:** 10.12669/pjms.306.5176

**Published:** 2014

**Authors:** Yue Jiang, Jun-Ping Tian, Hong Wang, Bu-Xing Chen, Feng-He Du

**Affiliations:** 1 Yue Jiang, Department of Cardiology, Beijing Tian Tan Hospital, Capital Medical University, Beijing, China.; 2 Jun-Ping Tian, Department of Cardiology, Beijing Tian Tan Hospital, Capital Medical University, Beijing, China.; 3 Hong Wang, Department of Endocrinology, Aerospace Central Hospital, Beijing, China.; 4 Bu-Xing Chen, Department of Cardiology, Beijing Tian Tan Hospital, Capital Medical University, Beijing, China.; 5 Feng-He Du, Department of Cardiology, Beijing Tian Tan Hospital, Capital Medical University, Beijing, China.

**Keywords:** Ambulatory electrocardiography, ST-segment deviation, Apnea hypopnea index, QT interval dispersion, Heart rate variability

## Abstract

***Background and Objective: ***The diagnostic value of ST-segment deviation detected by ambulatory electrocardiography (AECG) is controversial in identifying coronary artery disease (CAD) referred for coronary angiography (CAG). Recently, many parameters which evaluate CAD can be derived from AECG. Therefore, we aimed to investigate the diagnostic value of AECG in screening CAD referred for CAG when several parameters were combined.

***Methods: ***We studied the 104 chest pain inpatients. All patients received the CAG and AECG. A lumen diameter reduction of ≥ 50% was considered CAD according to CAG. The parameters derived from AECG included ST-segment deviation, apnea hypopnea index (AHI), QT interval dispersion (QTd) and heart rate variability (HRV). The diagnostic value of AECG in screening CAD was evaluated.

***Results: ***Of the 104 patients, 57 (54.8%) had CAD according to CAG. The sensitivity of ST-segment deviation in screening CAD was 64.9%; the specificity was 89.4%; and the Kappa value was 0.528. The sensitivity of at least three combined parameters including ST-segment deviation, AHI, QTd and HRV was 89.5%; the specificity was 87.2%; and the Kappa value was 0.767.

***Conclusion:*** AECG is very useful in screening CAD referred for CAG, especially while several parameters including ST-segment deviation, AHI, HRV and QTd are combined.

## INTRODUCTION

Coronary artery disease (CAD) is very common in most of the developing countries, including China^1^ and is projected to be the leading global cause of death and disability by 2020.^2^ Ambulatory electrocardiography (ECG) is used routinely for the detection of the myocardial ischemia in CAD patients. In the previous study, the ST-segment deviation detected from ambulatory ECG correlated well with other objective measurements used to define myocardial ischemia in CAD patients.^3^^-^^5^ However, the diagnostic significance of ambulatory ECG in CAD is suspected and controversial.^6^^,^^7^ Nair et al. showed that the ST-segment changes detected on ambulatory ECG were of limited value in identifying CAD and predicting the future cardiac events and death in unselected patients with chest pain.^7^ Recently, with the improvement of ambulatory ECG and computer technique, current ambulatory ECG equipment provides more detailed analysis of arrhythmias and ST-segment deviation, R-R intervals, heart rate variability (HRV), QRS-T morphology including late potentials, Q-T interval dispersion (QTd), T-wave alternans, and sleep apnea hypopnea index (AHI). Many studies have suggested that the HRV parameters correlate well with the angiographic extent of CAD and diminished HRV may be a useful prognostic indicator in CAD patients.^8^^-^^11^ There are also evidences indicating that sleep apnea may be an independent risk factor for CAD.^12^^-^^14^ Similarly, an increased QTd is a predictor of cardiac events in patients with heart disease.^15^

So in the present study, we aimed to investigate the diagnostic value of ambulatory ECG in the chest pain patients referred for coronary angiography, especially when several parameters derived from ambulatory ECG were combined.

## METHODS

***Patients: ***One hundred and four inpatients with chest pain in Department of Cardiology, Beijing Tian Tan Hospital, Capital Medical University between November 2008 and June 2009 were enrolled in this study. 

**Inclusion criteria:** The consecutive inpatients with chest pain referred for coronary angiography were included.

**Exclusion** **criteria** **: **Patients were excluded if they had any of the following conditions: (1) acute myocardial infarction, cardiomyopathy, or valvular heart disease, (2) abnormal electrocardial activity including cardiac conduction disturbances, rapid arrhythmia, left ventricular supervoltage, and pre-excitation syndrome, (3) ST-segment deviation due to abnormal electrolytes and drugs.

All the patients were instructed to continue all of their medications, including antianginal medications, statins, antiplatelet medications during the study. All the patients received 24-hour ambulatory ECG monitoring and coronary angiography. All the study participants underwent 24-hour ambulatory ECG monitoring within 72 hours after coronary angiography. The study protocol was approved by the ethics committees of the Beijing Tian Tan Hospital, Capital Medical University. Written informed consent was obtained from the patients or their relatives when the patients are prepared for coronary angiography.

***Routine*** ***examination: ***Venous blood samples were collected in the morning after the patients were admitted in hospital and overnight fast. Blood routine and biochemical parameters were measured by standard procedure. At the same time, ECG, chest X-ray and echocardiography were also performed.

***Ambulatory*** ***Electrocardiography: ***Continuous 24-h ambulatory ECG was performed using 12-lead ambulatory electrocardiogram, equipped with Impresario automatic analysis system (Del Mar Reynolds Medical Ltd, America). Subjects kept a detailed diary of the activities performed and symptoms experienced during the 24-hour monitoring period. Significant ST-segment shift was defined as either ≥1 mm ST elevation or ≥1 mm horizontal or downsloping ST-segment depression (60 to 80 ms from J-point) lasting ≥1 minute and separated from other episodes by ≥1 minute. If ST-segment depression always existed, obvious ST-segment shift was defined as ≥1 mm horizontal or down sloping ST-segment depression based on the reduced ST-segment. All episodes were verified by visual reading by an experienced observer blinded to clinical information. At the same time, effect of heart rate on ST-segment change should be noticed. The heart rates at the onset and the time of maximal ST-segment deviation were recorded for each individual episode. When heart rate changed in normal range, ST-segment shift was defined as 80 ms from J-point. When heart rate ≥ 120 beats per minute, ST-segment shift was defined as 50 ms from J-point. The physicians performing ECG analyses were blinded to the coronary angiographic finding and clinical characteristics of the patients.

Two time-domain measures of HRV were derived for each 24-hour period: (1) SDNN, which is the standard deviation of all RR intervals over 24-hour, and (2) pNN50, which is the percent difference between adjacent normal RR intervals >50 ms computed over the entire 24-hour ECG recording. The observed cutoff values of 24-hour measures of HRV, that is, SDNN <50 ms for highly depressed HRV, or SDNN <100 ms for moderately depressed HRV, pNN50 < 20% for obviously depressed HRV, are likely to be broadly applicable.^10^

The AHI was derived from ECG data using Lifescreen Apnea software. AHI ≥15/h was regarded as sleep apnea, while AHI＜15/h as nearly normal.^16^ Q-T interval and QTd was automatically analyzed according to ECG data from Impresario system. Continuous 8-10 lead Q-T intervals were measured. The QTd was the interlead difference between the maximal and minimal Q-T intervals.

***Coronary Angiography: ***Coronary angiography was undertaken using the Judkin’s technique by angiography machine (DFP2000A type 100mA, Japan). Multiple coronary angiograms were made in a variety of projections to significantly display the coronary arteries and their branches, and ensure the lesion visibility and accuracy. Coronary stenosis was assessed visually and interpreted by two experienced observers unaware of the results of the ambulatory ECG recordings. Significant CAD was defined as a ≥50% diameter reduction of a major coronary artery or branch seen on a coronary angiogram.

The patients were divided into the two groups according to coronary angiography: the CAD group (at least a ≥50% diameter reduction of a major coronary artery or branch) and control group (a lumen diameter reduction＜50%).

***Statistical Analysis: ***Continuous variables were expressed as mean±SD, while categorical variables were expressed as ratio or percentage. The Independent-Samples T Test was used when appropriate to compare differences for continuous variables. The comparison of ratio or percentage between both groups was performed by chi square test. Sensitivity, specificity, positive and negative predictive values, diagnostic accuracies, and Youden index were calculated. The Kappa Test was used to analyze the agreement of ambulatory ECG with coronary angiography. A two-tailed P-value less than 0.05 was considered to be statistically significant. All analyses were performed by SPSS for Windows Version 16.0.

## RESULTS

***The clinical characteristics of the study population: ***Based on the coronary angiogram, among the 104 patients enrolled in the present study, 57 (54.8%) were diagnosed as CAD patients and 47 (45.2%) non-CAD patients (control). The clinical characteristics of the two groups are shown in Table-I. The prevalence of diabetes mellitus, hypercholesterinemia, and smoking was obviously higher in CAD group as compared to control group (P＜0.05). The patients in CAD group also showed a higher body mass index than that in control group (P＜0.05). However, there were no significant differences in age, gender, or the prevalence of hypertension between the two groups.

***Diagnostic accuracy of the ambulatory ECG detected ST-segment deviation: ***Of the 57 CAD patients, 37 patients had ST-segment changes, and 20 patients had no ST-segment changes. Of the 47 control patients, 5 patients had ST-segment changes, and 42 patients had no ST-segment changes. The diagnostic accuracy of the ST-segment deviation detected by the ambulatory ECG monitoring in predicting the presence of CAD for all patients is given in Table-II. The sensitivity of ST-segment deviation in screening CAD was 64.9%; the specificity was 89.4%; and the Kappa value was 0.528.

***Diagnostic accuracy of combining several ambulatory ECG-derived parameters: ***Significant ST-segment deviation was defined as 1, while no ST-segment change as 0. The AHI≥15/h was defined as 1, while AHI＜15/h as 0. The SDNN＜100ms was defined as 1, while SDNN≥100ms as 0. The pNN50＜20% was defined as 1, while pNN50 ≥20% as 0. The QTd≥50ms was defined as 1, while QTd<50ms as 0.^17^ The above parameters were combined in parallel. The diagnostic accuracies of the several parameters combined detected by the ambulatory ECG in predicting the presence of CAD for all patients are given in Table-III. The Kappa value was the largest when three parameters were combined to detect CAD.

## DISCUSSION

In the present study we investigated the diagnostic value of ambulatory ECG in screening CAD referred for coronary angiography in chest pain patients. We found that the Kappa value of ambulatory ECG detected ST-segment change alone in predicting the presence of CAD was relatively low (0.528), however, the Kappa value of combining three ambulatory ECG-derived parameters in predicting the presence of CAD was significantly increased (0.767). Our study indicates that ambulatory ECG is very useful in screening CAD referred for coronary angiography.

**Table-I:** Baseline clinical characteristics.

**Variables**	**CAD group (n=57)**	**Control group (n=47)**	***p *** **value**
Age (years)	62.6±10.6	59.2±10.1	0.095
Gender (male/female)	40/17	30/17	0.497
Hypertension (%)	68.4	61.7	0.478
Diabetes mellitus (%)	54.4	17.0	0.000
Smoking (%)	63.2	42.6	0.036
Hypercholesterinemia (%)	84.2	55.3	0.002
Body mass index (kg/m^2^)	26.7±3.7	25.1±3.0	0.022

**Table-II:** Diagnostic accuracy of ST-segment deviation for detection of CAD.

**Diagnostic ** **characteristics**	**CAD**
Sensitivity	64.9%
Specificity	89.4%
Positive predictive value	88.1%
Negative predictive value	67.7%
Diagnostic accuracy	76.0%
Youden index	54.3%
Kappa value	0.528

**Table-III:** Diagnostic accuracy of several parameters combined for detection of CAD

**Diagnostic ** **characteristics**	**Two parameters**	**Three parameters**	**Four parameters**
Sensitivity	96.5%	89.5%	63.2%
Specificity	66.0%	87.2%	95.7%
Positive predictive value	77.5％	89.5％	94.7%
Negative predictive value	93.9％	87.2％	68.2%
Diagnostic accuracy	82.7%	88.5%	77.9%
Youden index	62.5%	76.7%	58.9%
Kappa value	0.641	0.767	0.569

Our study contradicts earlier data by Nair et al.^7^ reporting that the diagnostic accuracy of ambulatory ECG detected ST-segment deviation in predicting the presence of CAD was poor (33%), with a sensitivity of 19% and a specificity of 91%. In addition, Nair and his colleagues^6^ also found that the diagnostic and prognostic value of ischemia detected on ambulatory ECG monitoring were low in chest pain patients aged ＞70 years and those aged＜70 years. As compared with 3-lead ambulatory ECG in Nair’s study, we applied 12-lead ambulatory ECG to estimate myocardial ischemic. And several parameters combined for detection of CAD were adopted in our study other than ST-segment deviation alone.

In contrast, our finding was similar to Munoz del Romeral and coworkers’ report.^5^ However, the method to evaluate CAD in our study (coronary angiography) is more accurate than that in Munoz del Romeral’s (myocardial perfusion scintigraphy).

In our study, we found a higher diagnostic value of ambulatory ECG when several parameters derived from ambulatory ECG were combined and analyzed, reflecting possibly the different pathophysiological mechanisms of CAD.

Sleep apnea syndrome is a chronic sleep disorder. Many studies have suggested that it could be an independent risk factor for the cardiovascular diseases including myocardial infarction, heart failure, hypertension, and others.^12^-^14^ In our study, AHI can be derived from ambulatory ECG. The higher the AHI, the more severe is the sleep apnea. The HRV is used to describe variations of both instantaneous heart rate and RR intervals. With the availability of new, digital, high-frequency, 24-hour, multilead ECG recorders, HRV has the potential to provide additional valuable insight into physiological and pathological conditions and to enhance risk stratification.^9^,^10^ Many studies have suggested that HRV may be a strong and independent predictor of CAD and mortality after an acute myocardial infarction.^8^,^15^ The study showed that HRV was decreased when myocardial ischemia occurred.^9^ In addition, some studies found that QTd was prolonged in CAD patients^18^,^19^ and an increased QTd could be used to predict the future cardiac event.^15^ It is thus not surprising to see in the present study that the diagnostic value of ambulatory ECG was significantly improved when several parameters were combined, especially when three parameters as discussed above were combined in parallel. The Kappa value reached 76.7%, with a sensitivity of 89.5% and a specificity of 87.2%, which reflected a good consistency of ambulatory ECG monitoring with coronary angiography.

In this study, the patients in CAD group had a higher prevalence of diabetes, hypercholesterinemia, smoking, and body mass index than those in control group. However, the differences between the two groups had no decisive effect on our results.

This study has some limitations. Firstly, the study was cross-sectional and in need of further follow-up. Secondly, combination in parallel was adopted when several parameters were analyzed in the statistical analysis, which inevitably led to a higher sensitivity. Thirdly, the diagnostic value of three parameters combined was superior to that of four parameters in the present study. Maybe a number of other confounders not controlled for in the present study could influence the results of ambulatory ECG, and these were not controlled for.

## CONCLUSIONS

We found that diagnostic value of ambulatory ECG monitoring was significantly improved referred for coronary angiography when several parameters including ST-segment deviation, AHI, HRV and QTd were combined. Our study suggests that ambulatory ECG is very useful in screening CAD referred for coronary angiography, especially while several parameters are combined.
